# Ethyl 1-(2-hy­droxy­eth­yl)-2-phenyl-1*H*-benzimidazole-5-carboxyl­ate

**DOI:** 10.1107/S1600536810023639

**Published:** 2010-06-26

**Authors:** Nurasyikin Hamzah, Shafida Abd. Hamid, Aisyah Saad Abdul Rahim, Mohd Mustaqim Rosli, Hoong-Kun Fun

**Affiliations:** a Kuliyyah of Science, International Islamic University Malaysia, Jalan Sultan Ahmad Shah, Bandar Indera Mahkota, 25200 Kuantan, Pahang, Malaysia; b School of Pharmaceutical Sciences, Universiti Sains Malaysia, 11800 USM, Penang, Malaysia; c X-ray Crystallography Unit, School of Physics, Universiti Sains Malaysia, 11800 USM, Penang, Malaysia

## Abstract

There are two mol­ecules in the asymmetric unit of the title compound, C_18_H_18_N_2_O_3_. In each one, the benzimidazole ring system is essentially planar, with maximum deviations of 0.027 (1) and 0.032 (1)Å, and makes dihedral angles of 38.64 (6) and 41.48 (6)°, respectively, with the attached benzene rings. An intra­molecular C—H⋯O hydrogen bond is observed in each mol­ecule. The two independent mol­ecules are connected into a dimer by two inter­molecular O—H⋯N hydrogen bonds. In the crystal, mol­ecules form a two-dimensional layers parallel to (012) *via* weak inter­molecular C—H⋯O hydrogen bonds. In addition, weak π-π stacking inter­actions are observed with centroid–centroid distances of 3.5244 (12) and 3.6189 (12) Å.

## Related literature

For the applications of benzimidazole and its derivatives in the pharmaceutical and biological fields, see: Horton *et al.* (2003 ▶). These heterocycles can serve as mol­ecular scaffolds with versatile binding properties *via* modifications of their functional groups, see: DeSimone *et al.* (2004 ▶). For the biological activity of benzimidazole derivatives, see: Gowda *et al.* (2009 ▶); Tunçbilek *et al.* (2009 ▶); Achar *et al.* (2010 ▶). For related structures, see: Arumugam *et al.* (2010 ▶). For the stability of the temperature controller used for the data collection, see: Cosier & Glazer (1986 ▶).
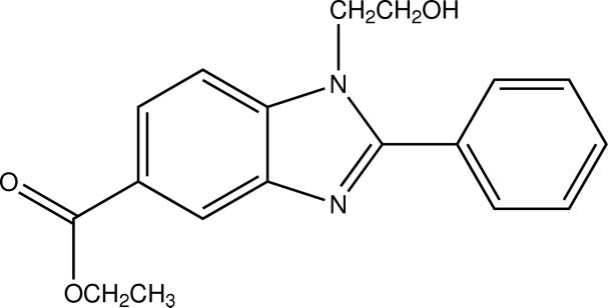


## Experimental

### 

#### Crystal data

C_18_H_18_N_2_O_3_
*M* *_r_* = 310.34
Triclinic, 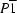

*a* = 8.997 (2) Å
*b* = 12.988 (3) Å
*c* = 15.030 (3) Å
α = 103.764 (6)°
β = 107.202 (6)°
γ = 102.929 (6)°
*V* = 1545.5 (6) Å^3^
*Z* = 4
Mo *K*α radiation
μ = 0.09 mm^−1^
*T* = 100 K
0.34 × 0.20 × 0.11 mm

#### Data collection

Bruker APEXII DUO CCD area-detector diffractometer
Absorption correction: multi-scan (*SADABS*; Bruker, 2009 ▶) *T*_min_ = 0.970, *T*_max_ = 0.990
34907 measured reflections
9838 independent reflections
6952 reflections with *I* > 2σ(*I*)
*R*_int_ = 0.051

#### Refinement

*R*[*F* ^2^ > 2σ(*F* ^2^)] = 0.051
*wR*(*F* ^2^) = 0.167
*S* = 1.04
9838 reflections
425 parameters
H atoms treated by a mixture of independent and constrained refinement
Δρ_max_ = 0.41 e Å^−3^
Δρ_min_ = −0.40 e Å^−3^

### 

Data collection: *APEX2* (Bruker, 2009 ▶); cell refinement: *SAINT* (Bruker, 2009 ▶); data reduction: *SAINT*; program(s) used to solve structure: *SHELXTL* (Sheldrick, 2008 ▶); program(s) used to refine structure: *SHELXTL*; molecular graphics: *SHELXTL*; software used to prepare material for publication: *SHELXTL* and *PLATON* (Spek, 2009 ▶).

## Supplementary Material

Crystal structure: contains datablocks global, I. DOI: 10.1107/S1600536810023639/lh5069sup1.cif

Structure factors: contains datablocks I. DOI: 10.1107/S1600536810023639/lh5069Isup2.hkl

Additional supplementary materials:  crystallographic information; 3D view; checkCIF report

## Figures and Tables

**Table 1:** Hydrogen-bond geometry (Å, °)

*D*—H⋯*A*	*D*—H	H⋯*A*	*D*⋯*A*	*D*—H⋯*A*
O1*A*—H1*OA*⋯N2*B*	0.81 (3)	1.96 (3)	2.7700 (19)	177 (3)
O1*B*—H1*OB*⋯N2*A*	0.98 (3)	1.89 (3)	2.8680 (18)	177 (2)
C2*B*—H2*BA*⋯O1*A*^i^	0.93	2.42	3.238 (2)	146
C12*B*—H12*B*⋯O1*B*^ii^	0.93	2.55	3.416 (2)	155
C13*A*—H13*A*⋯O1*A*	0.93	2.40	3.273 (2)	157
C13*B*—H13*B*⋯O1*B*	0.93	2.43	3.300 (2)	155
C15*A*—H15*B*⋯O2*A*^iii^	0.97	2.53	3.135 (2)	120
C17*B*—H17*D*⋯O2*B*^iv^	0.97	2.54	3.282 (2)	133

Symmetry codes: (i) 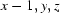; (ii) 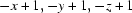; (iii) 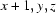; (iv) 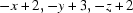.

**Fig. 1.**
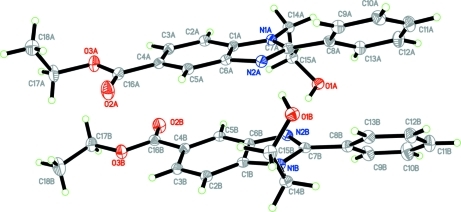
The molecular structure, showing 50% probability displacement ellipsoids and the atom-numbering scheme. Hydrogen atoms are shown as spheres of arbitrary radius.

**Fig. 2.**
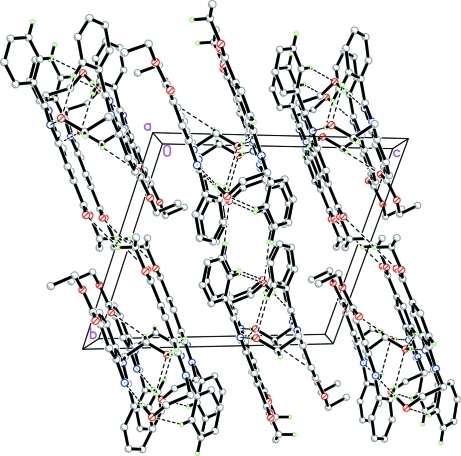
The crystal packing of (I) viewed along the a axis. Dashed lines indicate hydrogen bonds. H atoms not involved in the hydrogen bond interactions have been omitted for clarity.

## supplementary crystallographic information

### Comment

Benzimidazole and its derivatives are compounds which are well known in the pharmaceutical and biological fields (Horton *et al.*,  2003). These heterocycles can serve as molecular scaffolds with versatile binding properties *via* modifications of their functional groups (DeSimone *et al.*, 2004). Suitably substituted benzimidazole derivatives have been reported to show anti-tumor (Gowda *et al.*, 2009), antimicrobial (Tunçbilek *et al.*, 2009) and anti-inflammatory (Achar *et al.*, 2010) activities. On this basis, we report the structure of the title compound.

The asymmetric unit of (I) contains two molecules (Fig. 1) [A and B] with all geometrical parameters within normal ranges. For both molecules, the benzimidazole ring system (N1/N2/C1–C7) is essentially planar with a maximum deviation of 0.027 (1) and 0.032 (1)Å respectively for atom C7A and N1B. The dihedral angle between the benzimidazole ring system (N1/N2/C1–C7) and the attached benzene ring (C8–C13) is 38.64 (6) and 41.48 (6)° respectively for molecules A and B.

The two independent molecules are connected into a dimer by two intermolecular O—H···N hydrogen bonds (Table 1). In the crystal structure, molecules are connected by weak intermolecular C—H···O interactions (Table 1). These interactions form two-dimensional layers parallel to (0 1 2). In addition there are weak π···π stacking interactions within the asymmetric unit with distances of Cg1···Cg3 = 3.5244 (12) Å and Cg2···Cg4 = 3.6189 (12) Å; Cg1, Cg2, Cg3 and Cg4 and  are the centroids of N1A/N2A/C1A/C6A–C7A, C1A–C6A, N1B/N2B/C1B/C6B–C7B and C1B–C6B rings, respectively.

### Experimental

The title compound was synthesised by the addition  of sodium sulfite adduct of benzaldehyde (562 mg, 2.67 mmol) to a mixture of ethyl 3-amino-4-(2-hydroxylethylamino) benzoate  (300 mg, 1.33 mmol) in 0.5 mL of DMF. Subsequently, the mixture was irradiated at  403K in a microwave reactor for 2 min. After the reaction, the mixture was diluted with 30 mL of EtOAc and washed with 20 mL of H_2_O. The organic layer was collected, dried over Na_2_SO_4_ and the solvent was evaporated under pressure to afford the crude product. The product was recrystallised with hot EtOAc which was slowly evaporated to give a single block of clear crystal.

### Refinement

The H atoms attached to O1A and O1B were located in a difference map and refined isotropically. The remaining H atoms were positioned geometrically [C-H = 0.93, 0.96 or 0.97 Å] and were refined using a riding model, with Uiso(H) = xUeq(C), where x = 1.5 for methyl H and 1.2 for all other H atoms. A rotating group model was used for the methyl groups.

### Crystal data

**Table:**

C_18_H_18_N_2_O_3_	*Z* = 4
*M**_r_* = 310.34	*F*(000) = 656
Triclinic, *P*1	*D*_x_ = 1.334 Mg m^−^^3^
Hall symbol: -P 1	Mo *K*α radiation, λ = 0.71073 Å
*a* = 8.997 (2) Å	Cell parameters from 6508 reflections
*b* = 12.988 (3) Å	θ = 2.6–31.1°
*c* = 15.030 (3) Å	µ = 0.09 mm^−^^1^
α = 103.764 (6)°	*T* = 100 K
β = 107.202 (6)°	Block, colourless
γ = 102.929 (6)°	0.34 × 0.20 × 0.11 mm
*V* = 1545.5 (6) Å^3^	

### Data collection

**Table:**

Bruker APEXII DUO CCD area-detector diffractometer	9838 independent reflections
Radiation source: fine-focus sealed tube	6952 reflections with *I* > 2σ(*I*)
graphite	*R*_int_ = 0.051
φ and ω scans	θ_max_ = 31.1°, θ_min_ = 1.5°
Absorption correction: multi-scan (*SADABS*; Bruker, 2009)	*h* = −13→13
*T*_min_ = 0.970, *T*_max_ = 0.990	*k* = −18→18
34907 measured reflections	*l* = −21→21

### Refinement

**Table:**

Refinement on *F*^2^	Primary atom site location: structure-invariant direct methods
Least-squares matrix: full	Secondary atom site location: difference Fourier map
*R*[*F*^2^ > 2σ(*F*^2^)] = 0.051	Hydrogen site location: inferred from neighbouring sites
*wR*(*F*^2^) = 0.167	H atoms treated by a mixture of independent and constrained refinement
*S* = 1.03	*w* = 1/[σ^2^(*F*_o_^2^) + (0.0944*P*)^2^ + 0.2365*P*] where *P* = (*F*_o_^2^ + 2*F*_c_^2^)/3
9838 reflections	(Δ/σ)_max_ = 0.001
425 parameters	Δρ_max_ = 0.41 e Å^−^^3^
0 restraints	Δρ_min_ = −0.40 e Å^−^^3^

### Special details

**Table:**

Experimental. The crystal was placed in the cold stream of an Oxford Cyrosystems Cobra open-flow nitrogen cryostat (Cosier & Glazer, 1986) operating at 100.0 (1) K.
Geometry. All esds (except the esd in the dihedral angle between two l.s. planes) are estimated using the full covariance matrix. The cell esds are taken into account individually in the estimation of esds in distances, angles and torsion angles; correlations between esds in cell parameters are only used when they are defined by crystal symmetry. An approximate (isotropic) treatment of cell esds is used for estimating esds involving l.s. planes.
Refinement. Refinement of F^2^ against ALL reflections. The weighted R-factor wR and goodness of fit S are based on F^2^, conventional R-factors R are based on F, with F set to zero for negative F^2^. The threshold expression of F^2^ > 2sigma(F^2^) is used only for calculating R-factors(gt) etc. and is not relevant to the choice of reflections for refinement. R-factors based on F^2^ are statistically about twice as large as those based on F, and R- factors based on ALL data will be even larger.

### Fractional atomic coordinates and isotropic or equivalent isotropic displacement parameters (Å^2^)

**Table:**

	*x*	*y*	*z*	*U*_iso_*/*U*_eq_	
O1A	1.15213 (13)	0.95597 (9)	0.66787 (8)	0.0189 (2)	
O2A	0.54072 (14)	1.15419 (10)	0.96176 (9)	0.0311 (3)	
O3A	0.78042 (13)	1.28774 (9)	1.05882 (8)	0.0235 (2)	
N1A	1.02010 (14)	0.92733 (10)	0.82790 (8)	0.0147 (2)	
N2A	0.74895 (14)	0.84883 (10)	0.78666 (9)	0.0155 (2)	
C1A	0.96687 (16)	1.00752 (11)	0.87590 (10)	0.0148 (2)	
C2A	1.05169 (17)	1.11622 (12)	0.94178 (10)	0.0171 (3)	
H2AA	1.1641	1.1480	0.9596	0.020*	
C3A	0.95920 (17)	1.17389 (12)	0.97904 (10)	0.0171 (3)	
H3AA	1.0110	1.2462	1.0233	0.021*	
C4A	0.78835 (17)	1.12585 (12)	0.95159 (10)	0.0160 (3)	
C5A	0.70664 (17)	1.01725 (12)	0.88665 (10)	0.0161 (3)	
H5AA	0.5943	0.9853	0.8689	0.019*	
C6A	0.79750 (16)	0.95790 (12)	0.84909 (10)	0.0150 (3)	
C7A	0.88525 (16)	0.83316 (11)	0.77649 (10)	0.0149 (3)	
C8A	0.88701 (16)	0.72261 (11)	0.72397 (10)	0.0161 (3)	
C9A	0.78407 (17)	0.62979 (12)	0.73233 (11)	0.0192 (3)	
H9AA	0.7178	0.6404	0.7686	0.023*	
C10A	0.78010 (19)	0.52256 (13)	0.68722 (12)	0.0219 (3)	
H10A	0.7113	0.4616	0.6931	0.026*	
C11A	0.87878 (19)	0.50601 (13)	0.63319 (12)	0.0234 (3)	
H11A	0.8785	0.4342	0.6043	0.028*	
C12A	0.97797 (19)	0.59712 (13)	0.62236 (12)	0.0235 (3)	
H12A	1.0425	0.5858	0.5850	0.028*	
C13A	0.98171 (18)	0.70472 (12)	0.66663 (11)	0.0195 (3)	
H13A	1.0474	0.7651	0.6582	0.023*	
C14A	1.19033 (16)	0.94750 (12)	0.83426 (10)	0.0170 (3)	
H14A	1.2089	0.8763	0.8138	0.020*	
H14B	1.2633	0.9868	0.9023	0.020*	
C15A	1.23125 (17)	1.01573 (12)	0.77022 (10)	0.0178 (3)	
H15A	1.1993	1.0824	0.7850	0.021*	
H15B	1.3490	1.0392	0.7867	0.021*	
C16A	0.68862 (19)	1.18841 (12)	0.98976 (11)	0.0195 (3)	
C17A	0.6900 (2)	1.35453 (14)	1.09824 (13)	0.0270 (3)	
H17A	0.6001	1.3559	1.0441	0.032*	
H17B	0.7622	1.4307	1.1334	0.032*	
C18A	0.6227 (2)	1.30985 (16)	1.16688 (13)	0.0309 (4)	
H18A	0.5706	1.3591	1.1944	0.046*	
H18B	0.7105	1.3051	1.2190	0.046*	
H18C	0.5438	1.2369	1.1310	0.046*	
O1B	0.44589 (13)	0.70587 (9)	0.63176 (8)	0.0198 (2)	
O2B	0.98042 (13)	1.37266 (9)	0.85047 (9)	0.0261 (2)	
O3B	0.72718 (13)	1.37041 (9)	0.84618 (8)	0.0218 (2)	
N1B	0.56260 (14)	0.88812 (10)	0.55835 (8)	0.0146 (2)	
N2B	0.83526 (14)	0.96642 (10)	0.60695 (9)	0.0159 (2)	
C1B	0.60025 (16)	0.99572 (11)	0.61970 (10)	0.0144 (2)	
C2B	0.50052 (16)	1.05442 (12)	0.64814 (10)	0.0163 (3)	
H2BA	0.3873	1.0224	0.6255	0.020*	
C3B	0.57926 (17)	1.16295 (12)	0.71190 (10)	0.0170 (3)	
H3BA	0.5174	1.2055	0.7320	0.020*	
C4B	0.75098 (17)	1.21029 (11)	0.74702 (10)	0.0158 (3)	
C5B	0.84839 (16)	1.15126 (11)	0.71697 (10)	0.0155 (3)	
H5BA	0.9618	1.1830	0.7404	0.019*	
C6B	0.77088 (16)	1.04313 (11)	0.65068 (10)	0.0146 (2)	
C7B	0.70751 (16)	0.87512 (11)	0.55223 (10)	0.0150 (3)	
C8B	0.72348 (17)	0.77623 (12)	0.48846 (10)	0.0169 (3)	
C9B	0.83211 (19)	0.79407 (13)	0.43951 (12)	0.0235 (3)	
H9BA	0.8894	0.8666	0.4467	0.028*	
C10B	0.8546 (2)	0.70370 (15)	0.38006 (13)	0.0299 (4)	
H10B	0.9284	0.7160	0.3486	0.036*	
C11B	0.7677 (2)	0.59535 (14)	0.36748 (13)	0.0270 (3)	
H11B	0.7813	0.5352	0.3264	0.032*	
C12B	0.66018 (19)	0.57676 (13)	0.41618 (12)	0.0224 (3)	
H12B	0.6027	0.5040	0.4082	0.027*	
C13B	0.63813 (17)	0.66645 (12)	0.47681 (11)	0.0187 (3)	
H13B	0.5666	0.6536	0.5097	0.022*	
C14B	0.39725 (16)	0.80879 (12)	0.51552 (10)	0.0166 (3)	
H14C	0.3917	0.7454	0.4636	0.020*	
H14D	0.3204	0.8442	0.4864	0.020*	
C15B	0.34895 (17)	0.76807 (12)	0.59306 (11)	0.0187 (3)	
H15C	0.3599	0.8320	0.6463	0.022*	
H15D	0.2347	0.7220	0.5639	0.022*	
C16B	0.83472 (18)	1.32504 (12)	0.81937 (10)	0.0179 (3)	
C17B	0.7947 (2)	1.48136 (13)	0.91805 (12)	0.0243 (3)	
H17C	0.8708	1.5301	0.9000	0.029*	
H17D	0.8527	1.4787	0.9827	0.029*	
C18B	0.6526 (2)	1.52376 (16)	0.91910 (15)	0.0354 (4)	
H18D	0.6924	1.5977	0.9659	0.053*	
H18E	0.5786	1.4750	0.9374	0.053*	
H18F	0.5960	1.5256	0.8547	0.053*	
H1OA	1.061 (3)	0.9613 (18)	0.6519 (16)	0.033 (6)*	
H1OB	0.551 (3)	0.753 (2)	0.6837 (18)	0.048 (7)*	

### Atomic displacement parameters (Å^2^)

**Table:**

	*U*^11^	*U*^22^	*U*^33^	*U*^12^	*U*^13^	*U*^23^
O1A	0.0133 (5)	0.0260 (5)	0.0181 (5)	0.0069 (4)	0.0064 (4)	0.0070 (4)
O2A	0.0197 (5)	0.0316 (6)	0.0346 (7)	0.0082 (5)	0.0096 (5)	−0.0017 (5)
O3A	0.0230 (5)	0.0193 (5)	0.0255 (6)	0.0058 (4)	0.0114 (4)	0.0006 (4)
N1A	0.0137 (5)	0.0153 (5)	0.0152 (5)	0.0043 (4)	0.0061 (4)	0.0048 (4)
N2A	0.0133 (5)	0.0161 (5)	0.0162 (5)	0.0036 (4)	0.0058 (4)	0.0045 (4)
C1A	0.0155 (6)	0.0160 (6)	0.0139 (6)	0.0049 (5)	0.0064 (5)	0.0055 (5)
C2A	0.0146 (6)	0.0178 (6)	0.0168 (6)	0.0031 (5)	0.0047 (5)	0.0056 (5)
C3A	0.0178 (6)	0.0164 (6)	0.0152 (6)	0.0036 (5)	0.0055 (5)	0.0042 (5)
C4A	0.0169 (6)	0.0176 (6)	0.0142 (6)	0.0058 (5)	0.0059 (5)	0.0056 (5)
C5A	0.0140 (6)	0.0183 (6)	0.0158 (6)	0.0046 (5)	0.0053 (5)	0.0059 (5)
C6A	0.0148 (6)	0.0162 (6)	0.0135 (6)	0.0041 (5)	0.0048 (5)	0.0051 (5)
C7A	0.0143 (6)	0.0164 (6)	0.0144 (6)	0.0038 (5)	0.0056 (5)	0.0061 (5)
C8A	0.0141 (6)	0.0163 (6)	0.0166 (6)	0.0051 (5)	0.0038 (5)	0.0052 (5)
C9A	0.0179 (6)	0.0194 (7)	0.0204 (7)	0.0046 (5)	0.0077 (5)	0.0073 (5)
C10A	0.0207 (7)	0.0168 (7)	0.0262 (7)	0.0038 (5)	0.0072 (6)	0.0076 (6)
C11A	0.0223 (7)	0.0171 (7)	0.0274 (8)	0.0052 (6)	0.0080 (6)	0.0035 (6)
C12A	0.0230 (7)	0.0211 (7)	0.0277 (8)	0.0072 (6)	0.0137 (6)	0.0044 (6)
C13A	0.0190 (6)	0.0180 (7)	0.0215 (7)	0.0046 (5)	0.0091 (6)	0.0054 (5)
C14A	0.0131 (6)	0.0204 (7)	0.0187 (6)	0.0060 (5)	0.0068 (5)	0.0068 (5)
C15A	0.0131 (6)	0.0211 (7)	0.0196 (7)	0.0040 (5)	0.0071 (5)	0.0071 (5)
C16A	0.0227 (7)	0.0199 (7)	0.0163 (6)	0.0079 (5)	0.0077 (5)	0.0049 (5)
C17A	0.0333 (8)	0.0218 (7)	0.0321 (8)	0.0130 (6)	0.0202 (7)	0.0055 (6)
C18A	0.0329 (9)	0.0348 (9)	0.0279 (8)	0.0157 (7)	0.0129 (7)	0.0084 (7)
O1B	0.0212 (5)	0.0172 (5)	0.0204 (5)	0.0043 (4)	0.0072 (4)	0.0073 (4)
O2B	0.0185 (5)	0.0217 (6)	0.0302 (6)	0.0023 (4)	0.0064 (5)	0.0019 (5)
O3B	0.0196 (5)	0.0198 (5)	0.0242 (5)	0.0064 (4)	0.0088 (4)	0.0032 (4)
N1B	0.0121 (5)	0.0158 (5)	0.0157 (5)	0.0033 (4)	0.0054 (4)	0.0057 (4)
N2B	0.0138 (5)	0.0166 (6)	0.0178 (6)	0.0048 (4)	0.0059 (4)	0.0067 (4)
C1B	0.0130 (6)	0.0158 (6)	0.0145 (6)	0.0033 (5)	0.0046 (5)	0.0066 (5)
C2B	0.0127 (6)	0.0202 (7)	0.0169 (6)	0.0051 (5)	0.0060 (5)	0.0072 (5)
C3B	0.0160 (6)	0.0194 (7)	0.0177 (6)	0.0065 (5)	0.0077 (5)	0.0071 (5)
C4B	0.0168 (6)	0.0156 (6)	0.0148 (6)	0.0038 (5)	0.0060 (5)	0.0053 (5)
C5B	0.0131 (6)	0.0171 (6)	0.0163 (6)	0.0036 (5)	0.0046 (5)	0.0073 (5)
C6B	0.0127 (6)	0.0167 (6)	0.0161 (6)	0.0049 (5)	0.0057 (5)	0.0078 (5)
C7B	0.0133 (6)	0.0172 (6)	0.0163 (6)	0.0051 (5)	0.0055 (5)	0.0080 (5)
C8B	0.0154 (6)	0.0186 (7)	0.0173 (6)	0.0054 (5)	0.0064 (5)	0.0063 (5)
C9B	0.0247 (7)	0.0223 (7)	0.0304 (8)	0.0085 (6)	0.0165 (6)	0.0118 (6)
C10B	0.0358 (9)	0.0295 (8)	0.0367 (9)	0.0138 (7)	0.0267 (8)	0.0123 (7)
C11B	0.0291 (8)	0.0244 (8)	0.0288 (8)	0.0112 (6)	0.0143 (7)	0.0038 (6)
C12B	0.0209 (7)	0.0178 (7)	0.0270 (8)	0.0052 (5)	0.0089 (6)	0.0053 (6)
C13B	0.0170 (6)	0.0190 (7)	0.0212 (7)	0.0055 (5)	0.0081 (5)	0.0073 (5)
C14B	0.0117 (6)	0.0183 (6)	0.0177 (6)	0.0026 (5)	0.0043 (5)	0.0054 (5)
C15B	0.0160 (6)	0.0189 (7)	0.0224 (7)	0.0032 (5)	0.0099 (5)	0.0074 (5)
C16B	0.0191 (6)	0.0175 (6)	0.0177 (6)	0.0058 (5)	0.0069 (5)	0.0070 (5)
C17B	0.0266 (8)	0.0190 (7)	0.0251 (8)	0.0071 (6)	0.0107 (6)	0.0022 (6)
C18B	0.0384 (10)	0.0310 (9)	0.0467 (11)	0.0165 (8)	0.0253 (9)	0.0126 (8)

### Geometric parameters (Å, °)

**Table:**

O1A—C15A	1.4186 (18)	O1B—C15B	1.4109 (18)
O1A—H1OA	0.81 (2)	O1B—H1OB	0.98 (3)
O2A—C16A	1.2085 (19)	O2B—C16B	1.2069 (18)
O3A—C16A	1.3427 (18)	O3B—C16B	1.3484 (18)
O3A—C17A	1.4564 (18)	O3B—C17B	1.4463 (19)
N1A—C7A	1.3772 (18)	N1B—C7B	1.3779 (17)
N1A—C1A	1.3801 (17)	N1B—C1B	1.3815 (18)
N1A—C14A	1.4647 (18)	N1B—C14B	1.4563 (17)
N2A—C7A	1.3328 (18)	N2B—C7B	1.3306 (18)
N2A—C6A	1.3879 (18)	N2B—C6B	1.3900 (18)
C1A—C2A	1.4007 (19)	C1B—C2B	1.3953 (19)
C1A—C6A	1.4048 (19)	C1B—C6B	1.4037 (18)
C2A—C3A	1.386 (2)	C2B—C3B	1.384 (2)
C2A—H2AA	0.9300	C2B—H2BA	0.9300
C3A—C4A	1.413 (2)	C3B—C4B	1.408 (2)
C3A—H3AA	0.9300	C3B—H3BA	0.9300
C4A—C5A	1.393 (2)	C4B—C5B	1.3892 (19)
C4A—C16A	1.484 (2)	C4B—C16B	1.488 (2)
C5A—C6A	1.3889 (19)	C5B—C6B	1.3915 (19)
C5A—H5AA	0.9300	C5B—H5BA	0.9300
C7A—C8A	1.471 (2)	C7B—C8B	1.472 (2)
C8A—C13A	1.396 (2)	C8B—C9B	1.398 (2)
C8A—C9A	1.4030 (19)	C8B—C13B	1.402 (2)
C9A—C10A	1.384 (2)	C9B—C10B	1.390 (2)
C9A—H9AA	0.9300	C9B—H9BA	0.9300
C10A—C11A	1.387 (2)	C10B—C11B	1.386 (2)
C10A—H10A	0.9300	C10B—H10B	0.9300
C11A—C12A	1.389 (2)	C11B—C12B	1.388 (2)
C11A—H11A	0.9300	C11B—H11B	0.9300
C12A—C13A	1.386 (2)	C12B—C13B	1.390 (2)
C12A—H12A	0.9300	C12B—H12B	0.9300
C13A—H13A	0.9300	C13B—H13B	0.9300
C14A—C15A	1.522 (2)	C14B—C15B	1.5221 (19)
C14A—H14A	0.9700	C14B—H14C	0.9700
C14A—H14B	0.9700	C14B—H14D	0.9700
C15A—H15A	0.9700	C15B—H15C	0.9700
C15A—H15B	0.9700	C15B—H15D	0.9700
C17A—C18A	1.505 (2)	C17B—C18B	1.504 (2)
C17A—H17A	0.9700	C17B—H17C	0.9700
C17A—H17B	0.9700	C17B—H17D	0.9700
C18A—H18A	0.9600	C18B—H18D	0.9600
C18A—H18B	0.9600	C18B—H18E	0.9600
C18A—H18C	0.9600	C18B—H18F	0.9600
			
C15A—O1A—H1OA	106.6 (15)	C15B—O1B—H1OB	112.7 (14)
C16A—O3A—C17A	115.62 (13)	C16B—O3B—C17B	116.69 (12)
C7A—N1A—C1A	106.75 (11)	C7B—N1B—C1B	106.88 (11)
C7A—N1A—C14A	130.19 (12)	C7B—N1B—C14B	130.48 (12)
C1A—N1A—C14A	123.05 (12)	C1B—N1B—C14B	122.60 (11)
C7A—N2A—C6A	105.31 (11)	C7B—N2B—C6B	105.39 (11)
N1A—C1A—C2A	131.57 (13)	N1B—C1B—C2B	131.25 (12)
N1A—C1A—C6A	105.94 (12)	N1B—C1B—C6B	105.80 (12)
C2A—C1A—C6A	122.46 (13)	C2B—C1B—C6B	122.93 (13)
C3A—C2A—C1A	116.44 (13)	C3B—C2B—C1B	116.39 (13)
C3A—C2A—H2AA	121.8	C3B—C2B—H2BA	121.8
C1A—C2A—H2AA	121.8	C1B—C2B—H2BA	121.8
C2A—C3A—C4A	121.80 (13)	C2B—C3B—C4B	121.44 (13)
C2A—C3A—H3AA	119.1	C2B—C3B—H3BA	119.3
C4A—C3A—H3AA	119.1	C4B—C3B—H3BA	119.3
C5A—C4A—C3A	120.83 (13)	C5B—C4B—C3B	121.46 (13)
C5A—C4A—C16A	117.33 (13)	C5B—C4B—C16B	117.66 (12)
C3A—C4A—C16A	121.83 (13)	C3B—C4B—C16B	120.88 (13)
C6A—C5A—C4A	118.18 (13)	C4B—C5B—C6B	117.88 (12)
C6A—C5A—H5AA	120.9	C4B—C5B—H5BA	121.1
C4A—C5A—H5AA	120.9	C6B—C5B—H5BA	121.1
N2A—C6A—C5A	130.04 (13)	N2B—C6B—C5B	130.50 (12)
N2A—C6A—C1A	109.67 (12)	N2B—C6B—C1B	109.69 (12)
C5A—C6A—C1A	120.28 (13)	C5B—C6B—C1B	119.80 (12)
N2A—C7A—N1A	112.31 (12)	N2B—C7B—N1B	112.22 (12)
N2A—C7A—C8A	121.48 (12)	N2B—C7B—C8B	122.11 (12)
N1A—C7A—C8A	125.92 (12)	N1B—C7B—C8B	125.54 (12)
C13A—C8A—C9A	118.79 (13)	C9B—C8B—C13B	119.22 (13)
C13A—C8A—C7A	124.62 (13)	C9B—C8B—C7B	117.73 (13)
C9A—C8A—C7A	116.59 (12)	C13B—C8B—C7B	123.04 (12)
C10A—C9A—C8A	120.72 (14)	C10B—C9B—C8B	120.14 (14)
C10A—C9A—H9AA	119.6	C10B—C9B—H9BA	119.9
C8A—C9A—H9AA	119.6	C8B—C9B—H9BA	119.9
C9A—C10A—C11A	119.98 (14)	C11B—C10B—C9B	120.35 (15)
C9A—C10A—H10A	120.0	C11B—C10B—H10B	119.8
C11A—C10A—H10A	120.0	C9B—C10B—H10B	119.8
C10A—C11A—C12A	119.75 (14)	C10B—C11B—C12B	119.94 (15)
C10A—C11A—H11A	120.1	C10B—C11B—H11B	120.0
C12A—C11A—H11A	120.1	C12B—C11B—H11B	120.0
C13A—C12A—C11A	120.63 (14)	C11B—C12B—C13B	120.24 (14)
C13A—C12A—H12A	119.7	C11B—C12B—H12B	119.9
C11A—C12A—H12A	119.7	C13B—C12B—H12B	119.9
C12A—C13A—C8A	120.08 (13)	C12B—C13B—C8B	120.10 (13)
C12A—C13A—H13A	120.0	C12B—C13B—H13B	120.0
C8A—C13A—H13A	120.0	C8B—C13B—H13B	120.0
N1A—C14A—C15A	112.25 (11)	N1B—C14B—C15B	111.15 (11)
N1A—C14A—H14A	109.2	N1B—C14B—H14C	109.4
C15A—C14A—H14A	109.2	C15B—C14B—H14C	109.4
N1A—C14A—H14B	109.2	N1B—C14B—H14D	109.4
C15A—C14A—H14B	109.2	C15B—C14B—H14D	109.4
H14A—C14A—H14B	107.9	H14C—C14B—H14D	108.0
O1A—C15A—C14A	113.10 (12)	O1B—C15B—C14B	112.51 (11)
O1A—C15A—H15A	109.0	O1B—C15B—H15C	109.1
C14A—C15A—H15A	109.0	C14B—C15B—H15C	109.1
O1A—C15A—H15B	109.0	O1B—C15B—H15D	109.1
C14A—C15A—H15B	109.0	C14B—C15B—H15D	109.1
H15A—C15A—H15B	107.8	H15C—C15B—H15D	107.8
O2A—C16A—O3A	123.39 (14)	O2B—C16B—O3B	123.64 (14)
O2A—C16A—C4A	123.88 (14)	O2B—C16B—C4B	124.81 (14)
O3A—C16A—C4A	112.73 (13)	O3B—C16B—C4B	111.55 (12)
O3A—C17A—C18A	112.42 (14)	O3B—C17B—C18B	106.85 (14)
O3A—C17A—H17A	109.1	O3B—C17B—H17C	110.4
C18A—C17A—H17A	109.1	C18B—C17B—H17C	110.4
O3A—C17A—H17B	109.1	O3B—C17B—H17D	110.4
C18A—C17A—H17B	109.1	C18B—C17B—H17D	110.4
H17A—C17A—H17B	107.9	H17C—C17B—H17D	108.6
C17A—C18A—H18A	109.5	C17B—C18B—H18D	109.5
C17A—C18A—H18B	109.5	C17B—C18B—H18E	109.5
H18A—C18A—H18B	109.5	H18D—C18B—H18E	109.5
C17A—C18A—H18C	109.5	C17B—C18B—H18F	109.5
H18A—C18A—H18C	109.5	H18D—C18B—H18F	109.5
H18B—C18A—H18C	109.5	H18E—C18B—H18F	109.5
			
C7A—N1A—C1A—C2A	176.50 (14)	C7B—N1B—C1B—C2B	177.03 (14)
C14A—N1A—C1A—C2A	−4.2 (2)	C14B—N1B—C1B—C2B	−5.3 (2)
C7A—N1A—C1A—C6A	−1.25 (14)	C7B—N1B—C1B—C6B	−1.51 (14)
C14A—N1A—C1A—C6A	178.06 (12)	C14B—N1B—C1B—C6B	176.20 (11)
N1A—C1A—C2A—C3A	−178.13 (13)	N1B—C1B—C2B—C3B	179.89 (13)
C6A—C1A—C2A—C3A	−0.7 (2)	C6B—C1B—C2B—C3B	−1.8 (2)
C1A—C2A—C3A—C4A	−0.4 (2)	C1B—C2B—C3B—C4B	−1.0 (2)
C2A—C3A—C4A—C5A	1.0 (2)	C2B—C3B—C4B—C5B	1.9 (2)
C2A—C3A—C4A—C16A	−178.65 (13)	C2B—C3B—C4B—C16B	−176.95 (13)
C3A—C4A—C5A—C6A	−0.5 (2)	C3B—C4B—C5B—C6B	0.0 (2)
C16A—C4A—C5A—C6A	179.14 (12)	C16B—C4B—C5B—C6B	178.85 (12)
C7A—N2A—C6A—C5A	−178.09 (14)	C7B—N2B—C6B—C5B	178.20 (14)
C7A—N2A—C6A—C1A	0.21 (15)	C7B—N2B—C6B—C1B	−0.70 (15)
C4A—C5A—C6A—N2A	177.63 (13)	C4B—C5B—C6B—N2B	178.57 (13)
C4A—C5A—C6A—C1A	−0.5 (2)	C4B—C5B—C6B—C1B	−2.63 (19)
N1A—C1A—C6A—N2A	0.67 (15)	N1B—C1B—C6B—N2B	1.40 (14)
C2A—C1A—C6A—N2A	−177.34 (12)	C2B—C1B—C6B—N2B	−177.30 (12)
N1A—C1A—C6A—C5A	179.17 (12)	N1B—C1B—C6B—C5B	−177.64 (12)
C2A—C1A—C6A—C5A	1.2 (2)	C2B—C1B—C6B—C5B	3.7 (2)
C6A—N2A—C7A—N1A	−1.05 (15)	C6B—N2B—C7B—N1B	−0.29 (15)
C6A—N2A—C7A—C8A	173.07 (12)	C6B—N2B—C7B—C8B	175.92 (12)
C1A—N1A—C7A—N2A	1.49 (15)	C1B—N1B—C7B—N2B	1.17 (15)
C14A—N1A—C7A—N2A	−177.76 (12)	C14B—N1B—C7B—N2B	−176.30 (12)
C1A—N1A—C7A—C8A	−172.31 (12)	C1B—N1B—C7B—C8B	−174.89 (12)
C14A—N1A—C7A—C8A	8.4 (2)	C14B—N1B—C7B—C8B	7.6 (2)
N2A—C7A—C8A—C13A	145.39 (14)	N2B—C7B—C8B—C9B	−37.4 (2)
N1A—C7A—C8A—C13A	−41.3 (2)	N1B—C7B—C8B—C9B	138.34 (15)
N2A—C7A—C8A—C9A	−33.94 (19)	N2B—C7B—C8B—C13B	141.16 (14)
N1A—C7A—C8A—C9A	139.34 (14)	N1B—C7B—C8B—C13B	−43.1 (2)
C13A—C8A—C9A—C10A	2.0 (2)	C13B—C8B—C9B—C10B	0.0 (2)
C7A—C8A—C9A—C10A	−178.61 (13)	C7B—C8B—C9B—C10B	178.61 (15)
C8A—C9A—C10A—C11A	0.1 (2)	C8B—C9B—C10B—C11B	1.1 (3)
C9A—C10A—C11A—C12A	−1.8 (2)	C9B—C10B—C11B—C12B	−1.4 (3)
C10A—C11A—C12A—C13A	1.3 (2)	C10B—C11B—C12B—C13B	0.6 (3)
C11A—C12A—C13A—C8A	0.9 (2)	C11B—C12B—C13B—C8B	0.5 (2)
C9A—C8A—C13A—C12A	−2.5 (2)	C9B—C8B—C13B—C12B	−0.8 (2)
C7A—C8A—C13A—C12A	178.16 (14)	C7B—C8B—C13B—C12B	−179.33 (13)
C7A—N1A—C14A—C15A	102.15 (16)	C7B—N1B—C14B—C15B	104.85 (16)
C1A—N1A—C14A—C15A	−76.99 (16)	C1B—N1B—C14B—C15B	−72.28 (16)
N1A—C14A—C15A—O1A	−69.79 (15)	N1B—C14B—C15B—O1B	−64.92 (15)
C17A—O3A—C16A—O2A	−0.9 (2)	C17B—O3B—C16B—O2B	−1.5 (2)
C17A—O3A—C16A—C4A	178.92 (12)	C17B—O3B—C16B—C4B	178.76 (12)
C5A—C4A—C16A—O2A	−6.3 (2)	C5B—C4B—C16B—O2B	3.6 (2)
C3A—C4A—C16A—O2A	173.36 (14)	C3B—C4B—C16B—O2B	−177.50 (14)
C5A—C4A—C16A—O3A	173.93 (12)	C5B—C4B—C16B—O3B	−176.65 (12)
C3A—C4A—C16A—O3A	−6.43 (19)	C3B—C4B—C16B—O3B	2.24 (19)
C16A—O3A—C17A—C18A	73.83 (18)	C16B—O3B—C17B—C18B	167.61 (13)

### Hydrogen-bond geometry (Å, °)

**Table:**

*D*—H···*A*	*D*—H	H···*A*	*D*···*A*	*D*—H···*A*
O1A—H1OA···N2B	0.81 (3)	1.96 (3)	2.7700 (19)	177 (3)
O1B—H1OB···N2A	0.98 (3)	1.89 (3)	2.8680 (18)	177 (2)
C2B—H2BA···O1A^i^	0.93	2.42	3.238 (2)	146
C12B—H12B···O1B^ii^	0.93	2.55	3.416 (2)	155
C13A—H13A···O1A	0.93	2.40	3.273 (2)	157
C13B—H13B···O1B	0.93	2.43	3.300 (2)	155
C15A—H15B···O2A^iii^	0.97	2.53	3.135 (2)	120
C17B—H17D···O2B^iv^	0.97	2.54	3.282 (2)	133

Symmetry codes: (i) *x*−1, *y*, *z*; (ii) −*x*+1, −*y*+1, −*z*+1; (iii) *x*+1, *y*, *z*; (iv) −*x*+2, −*y*+3, −*z*+2.
